# Design of Moving Target Detection System Using Lightweight Deep Learning Model and Its Impact on the Development of Sports Industry

**DOI:** 10.1155/2022/3252032

**Published:** 2022-07-20

**Authors:** Hongling Zhang, Yifei Zheng

**Affiliations:** ^1^Physical Institute, Yan'an University, Yan'an 716000, Shaanxi, China; ^2^Physical Department, Chang'an University, Xi'an 710064, Shaanxi, China

## Abstract

The intelligent tracking and detection of athletes' actions and the improvement of action standardization are of great practical significance to reducing the injury caused by sports in the sports industry. For the problems of nonstandard movement and single movement mode, this exploration takes the video of sports events as the object and combines it with the video general feature extraction of convolutional neural network (CNN) in the field of deep learning and the filtering detection algorithm of motion trajectory. Then, a target detection and tracking system model is proposed to track and detect targets in sports in real-time. Moreover, through experiments, the performance of the proposed system model is analyzed. After testing the detection quantity, response rate, data loss rate, and target detection accuracy of the model, the results show that the model can track and monitor 50 targets with a loss rate of 3%, a response speed of 4 s and a target detection accuracy of 80%. It can play an excellent role in sports events and postgame video analysis, and provide a good basis and certain design ideas for the goal tracking of the sports industry.

## 1. Introduction

Recently, rich achievements have been made in researching electronic information detection technology [1]. Various detection technologies have played an important role in multiple fields, such as traffic guidance, product detection, and information monitoring for private vehicles in daily life [2], dynamic monitoring of processes in various production activities [3], or matching and identification of private information in personal goods [4]. Among them, in the field of sports, people often have some sports injuries due to nonstandard movements or improper sports plans in the sports process, which makes it impossible for people to achieve the goal of improving physical fitness. When using traditional methods to analyze the trajectory or mode of motion, people must rely on massive analysis data, which needs high computing power. However, complex computing will affect the operational efficiency of the system. This problem has perplexed scholars. Similarly, this problem is not reasonably solved, resulting in a bottleneck in video tracking research. The emergence of intelligent algorithms makes human motion tracking and detection possible. As one of the artificial intelligence algorithms, deep learning (DL) or machine learning can effectively extract the characteristics of specific targets through autonomous learning to achieve the effect of tracking or monitoring specific targets [5]. DL is a technology that can screen specific projects from a large amount of data through similar characteristics, which plays a good auxiliary role in target tracking and monitoring [6].

As an efficient detection technology for detected images, the artificial intelligence algorithm named DL has been studied by multiple scholars. Fu et al. believed that some current target detection algorithms could not meet accuracy and speed requirements. Hence, the advantages of DL in big data feature learning should be fully used to suppress the weight of invalid features and improve the detection accuracy [7]. Remote sensing tracking image technology is a technology that can monitor multiple targets in real-time at the same time. Its performance is better than ordinary image monitoring. Xuan et al. used a filter to solve the problem of tracking failure and occlusion when the moving target was partially or completely tracked, and they could continuously monitor specific events by providing high-time resolution remote sensing images [8]. DL and neural networks are also auxiliary technologies commonly used in target detection technology. Lee and Lee proposed a very effective method based on the DL framework for real-time airborne computing capability on small unmanned aerial vehicles with limited space, proving the proposed algorithm's effectiveness [9]. An et al. proposed an improved rotating bounding box coding scheme with a convolutional neural network (CNN) to alleviate the imbalance between detection targets and found that it could produce better results than using just one alone [10]. Target detection technology has played a significant role in many industries. For example, to improve the effect of athlete's motion recognition based on image recognition technology, Zhang and Zhang combined with the research status of image recognition, studied athlete's motion recognition from the perspective of image processing, and used morphological operators to deal with noise and hole phenomena in foreground images [11]. Through the analysis of the research of the above scholars, it is found that many scholars apply artificial intelligence algorithms such as DL to the detection of specific targets. The specific application fields are more extensive, but the framework structure of intelligent algorithms is more cumbersome. Therefore, on the whole, the research prospect of target detection technology for real-time monitoring of athletes or other events in the sports industry is quite good.

The research innovation is to start with lightweight DL, and use the error back propagation algorithm in lightweight DL as the technical core of this exploration. The application of DL technology in sports target tracking technology is studied, and the application of this real-time tracking technology in the sports industry is explored. First, the related concepts of DL and development and application are described. After that, the principle of target tracking technology will be described. Next, the target management and collection module of sports are analyzed. Finally, the stability of tracking technology is tested to judge whether tracking technology can run stably in sports. It is hoped that a relevant experimental basis can be provided for the tracking and monitoring technology of sports goals in the follow-up sports field.

## 2. Construction and Application Analysis of Moving Target Detection System Based on Lightweight DL

### 2.1. General Video Feature Extraction Method Based on DL

**Figure 1:**
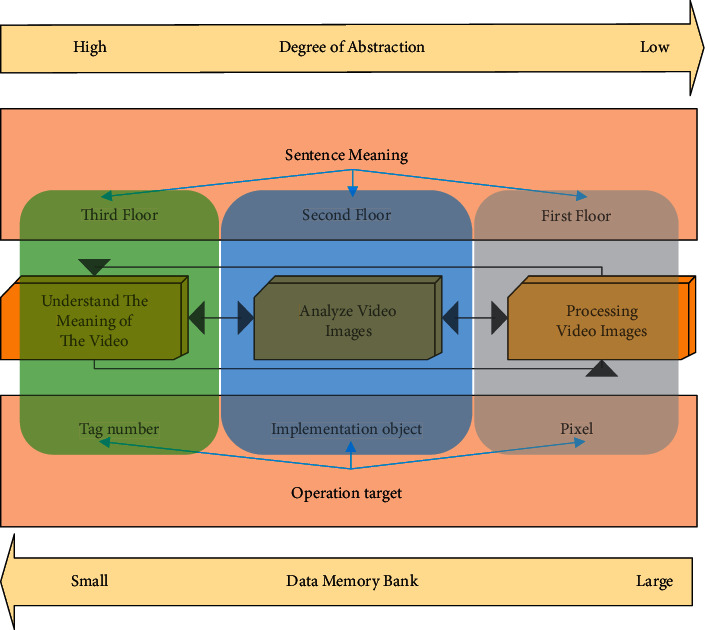
Video image processing process.

First, how to learn a general image feature extractor based on a cascade noise reduction autoencoder from massive auxiliary data in an unsupervised and off-line way is introduced in detail. A cascading noise reduction autoencoder is obtained by stacking several single-layer noise reduction autoencoder networks [12].  Figure 1 shows the general process of typical video processing.

Figure 1 shows that the video image processing process mainly includes video image processing, analysis, and understanding. It is the display of the abstraction degree of image content from low to high, and obtains the image from specific pixels to understand the symbols and motion actions in the image, which is also the understanding of the image from low level to high level.

A cascading noise reduction autoencoder can be obtained by training and stacking the noise reduction autoencoder layer-by-layer. This layer-by-layer training process is called the pretraining process. The training results will be used as the initial value of the feedforward neural network when the cascade noise reduction autoencoder is spread into a multilayer feedforward neural network. According to the specific follow-up tasks (usually supervised learning tasks), the parameters of this feedforward neural network will be fine-tuned by an error feedback algorithm.

**Figure 2:**
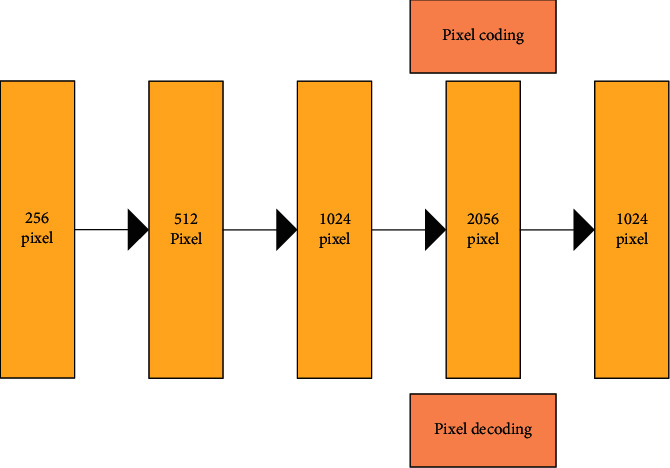
Encoder structure.

The samples of the tracking target in each frame are represented by the image pixels marked by a rectangular bounding box. Here, the gray image block with a size of 32 × 32 is used. After the two-dimensional image block is pulled into a vector with a size of 1024, it is used as the input of the neural network [13].  Figure 2 is a structural diagram of an encoder.

Figure 2 shows that in the normal target tracking task, the sample of the tracked target in each frame is represented by an image block marked by a rectangular bounding box. A gray image block with a size of 32 × 32 is taken as an example. After the two-dimensional image block is pulled into a vector, the size is 1024. Then, it is used as the input of the neural network. For the first hidden layer, this exploration uses the super complete configuration and uses the number of hidden nodes twice the size of the input layer. The target is represented by a sparse combination of a set of super complete redundant bases. The number of nodes in the subsequent hidden layer decreases in turn, and a bottleneck layer is formed in the third hidden layer.

Target tracking involves the processing and matching of the state and motion trajectory of the target object. The state of an object is to describe the trajectory state at a certain time in an 8-dimensional space. The matching of motion trajectory is to use the Mahalanobis distance of the positioning position predicted in the filter by the detection result and tracking result to express its degree [14]. The equation is as follows:

(1) $$\begin{array}{c} d^{\left(1\right)}\left(i,j\right)={\left(d_{j}-y_{i}\right)}^{T}S_{i}^{-1}\left(d_{j}-y_{i}\right), \end{array}$$

*y*_*i*_ is the predicted observation of the trajectory, *d*_*j*_ is the result state of the *j*-th, and *S*_*i*_ is the covariance matrix obtained through the filter.

The apparent matching degree is the phenomenon of data deformation after only using the Mahalanobis distance to predict. If the camera moves, it may cause the failure of the Mahalanobis distance prediction, which needs to be remedied to the apparent degree.

The development of DL is inseparable from the support of open-source software frameworks. At present, most neural network models need many samples for training. With a neural network as an example, multiple matrix operations are needed to calculate many floating-point numbers. The traditional central processing unit cannot deal with this situation efficiently. The graphics processing unit (GPU) is very suitable for parallel computing of large-scale matrices [15]. In recent years, the vigorous development of GPU has provided “weapons” for DL. The GPU chip with stronger computing power, larger video memory, and higher throughput can support the neural network model with deepening depth, increasing training data scale and increasing computational complexity.  Table 1 shows the widely used DL image processing methods [16].

There are relatively more datasets of scene classification tasks in the above datasets. However, the datasets for remote sensing image target detection are few, which is also a matter of course, because target detection requires many images to select boxes and label categories, which is much more cumbersome than classification tasks and consumes a lot of manpower and time.

### 2.2. Target Tracking Technology Based on CNN

**Figure 3:**
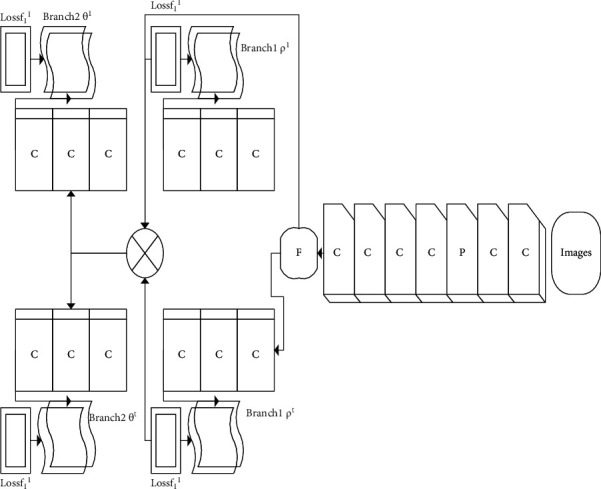
Attitude recognition process of moving target.

The application of CNN in the field of machine vision has achieved fruitful results. Research has shown that multilayer CNN can learn different levels of feature abstraction of the original input image [17], and has excellent learning ability and generalization ability for various machine vision tasks. Given the various advantages of CNN in capturing visual features and generalization ability [18, 19], a multilayer CNN is used in this section to model the appearance of various tracking targets.  Figure 3 is a structural process of attitude recognition of moving objects based on a neural network.

Figure 3 shows that for a typical online target tracking task, the initial position of the tracking target is usually marked in the form of a rectangular bounding box in the first frame of the image sequence. When using CNN to model the target's appearance, it is necessary to extract positive and negative samples according to the annotation in the first frame so that the network can be initialized and trained to convergence. Therefore, CNN takes a little time to initialize the first frame to get better initial model parameters.

The traditional target detection method is mainly based on a machine learning algorithm [20], which is mainly divided into two processes: training and testing. In the training process, the training set contains both positive and negative samples. The positive samples are images with targets, while the negative samples are images without targets. A trained model is finally obtained through feature extraction, feature dimensionality reduction, and classifier training. The test set is some actual test images, which may contain multiple targets or just background. The test needs to be carried out through various project areas like sliding windows, which become candidate boxes [19].  Table 2 shows the three core regional features of general target tracking.

There are two important problems with traditional target detection algorithms. First, the complexity of the region selection algorithm is high, the resulting window is easy to be redundant, and the detection speed is very slow. Second, the features designed by hand are not robust [21], and it is difficult to identify all kinds of objects with one or more features.

### 2.3. Target Detection Requirements for Sports Events

A moving object detection and tracking system for sports video needs to carry out unified detection and tracking management of moving objects in sports video. How to analyze and manage sports video conveniently is a problem that needs to be solved in the current application management system [22]. Therefore, the sports video moving target detection and tracking system studied here needs to provide users with operational feasibility.

**Figure 4:**
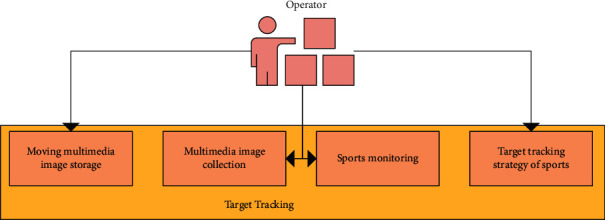
Sports target tracking process.

The users of the sports video moving target detection and tracking system mainly include system video managers, ordinary users, video analysts, and collectors. Users access the external network through Ethernet and connect to the router through the firewall. Then, the router is connected to the gateway. The gateway is connected with the application system server and data access server through Ethernet. In this way, the interaction process between the client and the server is completed.  Figure 4 is a tracking process when detecting a sports target.

**Figure 5:**
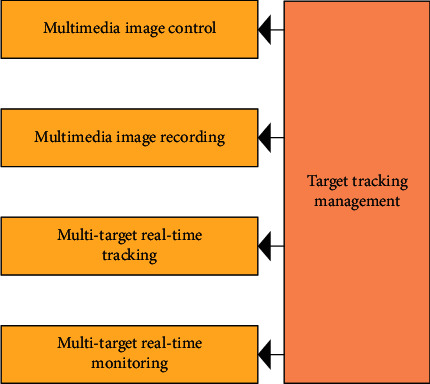
Basic functions of moving target tracking.

The system is a sports video moving target detection and tracking system with simple function, practicality, and operation. It mainly aims to solve specific problems in sports video management. In the system development and design, the modular development method is adopted, that is, the whole system is divided into multiple relatively independent functions according to the functions. The sports video moving target detection and tracking system is designed in detail according to the above requirements. It is mainly composed of sports video management function, video acquisition management function, target detection management function and target tracking management function.  Figure 5 is the basic functional composition of sports project management.

In the whole management system, sports video data management is very important. All video information is streaming media, so it is necessary to carry out unified collection, tracking, and detection, and then get the analysis data to assist athletes' training.

**Figure 6:**
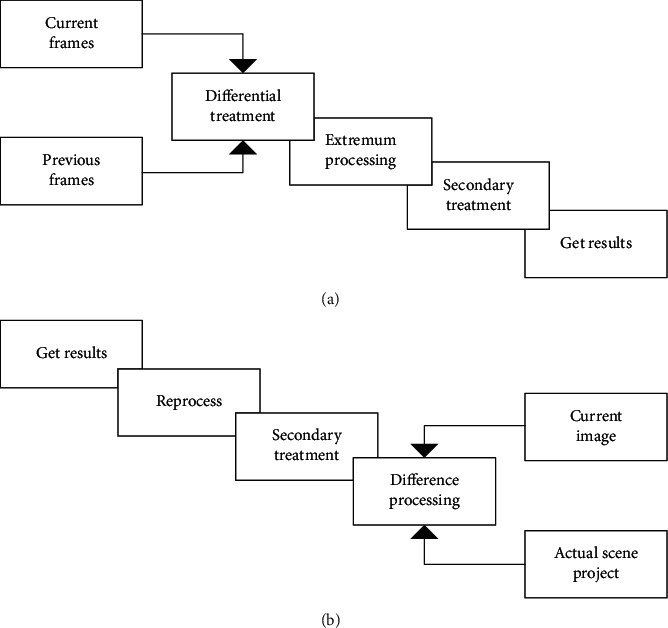
Target tracking technology ((a) is interframe difference processing method, (b) is background subtraction processing method).

At present, there are two main moving target detection algorithms: the interframe difference processing method and the background subtraction processing method. The two are generally used for moving target detection in a static background [23]. The optical flow method can be used for both static and dynamic backgrounds. Besides, various algorithms have their own advantages, disadvantages, and application scope.  Figure 6 shows the commonly used target tracking technology.

The operators of the video information management function are mainly video administrators, video personnel, and acquisition personnel. The sports video management function is mainly to maintain sports video, such as adding, modifying, deleting, and querying. Among them, the video administrator has the operation authority over all subfunctions in this function, and other personnel only have the operation authority over the video viewing function.

### 2.4. Management Module Requirements of Sports Target Detection

The use of this function is as follows: first, the collector collects the video of an athlete's sports process or training process. Then, the collected video data is maintained and processed. The video manager completes the operation of adding the video's basic information by inputting the video's basic information. Basic video information includes: video shot, video number, video name, video acquisition time, video size, acquisition method, video content information, and collector information.

In the system, any video is linked by shots, so shots are the basic unit of video retrieval. The video sequence is mainly composed of video shots. The content of each shot occurs in one scene, or one scene can be listed in multiple shots. Video sequence processing is to process the objects or images contained in the lens. Video data mainly includes story units, scenes, shots, and frames [24].

The user needs are basically mastered through the demand analysis of sports video moving target detection and tracking systems in the previous section. This section will design the system prototype based on the analysis. The sports video moving object detection and tracking system mainly has a three-layer structure, namely the application layer, data access layer, and data connection layer.  Table 3 shows the specific requirements of these three layers.

The sports video management module mainly includes video maintenance and management functions, video upload functions, video download functions, and video retrieval functions. The video acquisition module includes video input and initialization, acquiring camera video, reading in AVI files and acquiring sequence images. The target detection management module mainly includes target detection, establishing a target model, updating a target model, and displaying detection results. The target tracking management module includes the establishment of a tracking target instance, the establishment of a target template, target matching processing, loss tracking processing, and parameter result display.

The sports target detection technology based on this can monitor the sports target in real-time. In the next research process, the system's performance will be tested to judge the universality and excellence of target tracking technology.

## 3. Results

### 3.1. Traffic Speed and Response Performance of Sports Target Detection System

**Figure 7:**
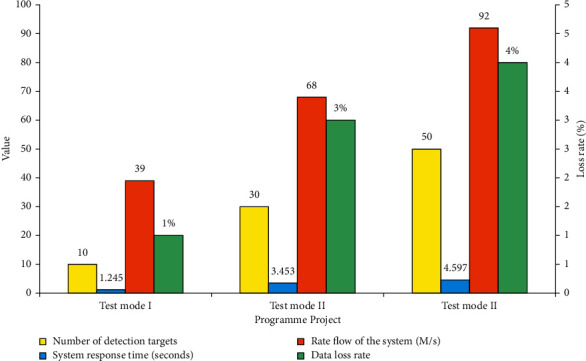
Performance detection of the target detection system.

The video retrieval function is tested in the submodule of sports video management. The administrator logs in to the system, clicks on sports video management to enter the interface of sports video management, and clicks video retrieval to enter the interface of video retrieval. In this link, the system's stability and real-time error correction performance are tested by testing the response rate, time, server performance, interface traffic, and data loss rate of the target detection system under the condition of a different number of users. In the test, it is essential to continuously improve the number of concurrent users, detect the network speed and traffic, and record the response data changes.  Figure 7 shows the results.

The data in Figure 7 shows that three methods are used to test the target tracking system in this section. In the first test scheme, there are 10 tracking targets to be detected, and the response time of the target tracking system is 1.245 seconds. The rate of flow of the system is 39 Mb/s, and the loss value of data is only 1%. It reveals that the system's response time is short, which can meet the needs of detection. Moreover, the system speed can also ensure a better standard. In the second test scheme, there are 30 tracking targets to be detected. The response time of the target tracking system is 3.453 seconds, the rate of flow of the system is 68 Mb/s, and the data loss rate is 3%. It suggests that the system's response time has increased, but the growth rate is not high, and the system rate also ensures a growth rate of nearly 1.8 times. In the third test scheme, there are 50 tracking targets to be detected. The response time of the target tracking system is 4.597 seconds, the rate of flow of the system is 92 mb/s, and the data loss rate is only 4%. It reveals that after adding the detection target, the response rate of the system slows down. However, the overall rate increases greatly, and the growth rate of the data loss rate is very small, indicating that the performance of the system is very excellent and can detect the target well in a stable state.

### 3.2. Traffic Speed and Response Performance of Sports Target Detection System

**Figure 8:**
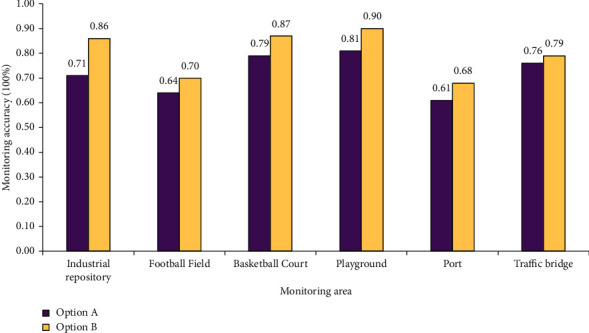
Data of system monitoring accuracy results.

In the last section, the performance of the target tracking system is verified, which mainly tests the performance values of the system in three aspects: system response time, response rate, and data loss rate when detecting different numbers of targets. Through the discussion of the results, the premise of the high stability of the system model is determined. Therefore, this section will test the real-time monitoring accuracy of the tracking system. Two schemes are tested. Scheme A determines 30 detection targets and Scheme B determines 50 detection targets. The results are analyzed by testing the monitoring accuracy, as shown in Figure 8.

The target tracking and monitoring accuracy value in Figure 8 shows that the monitoring accuracy of the system can be guaranteed to be more than 60% for 30 or 50 people. The monitoring accuracy in the industrial storage area is 71% for 30 people and 86% for 50 people. On the football field, the monitoring accuracy is 64% for 30 people and 70% for 50 people. In the basketball court, the monitoring accuracy is 79% for 30 people and 87% for 50 people. In the playground, the monitoring accuracy is 81% for 30 people and 90% for 50 people. In the port, the monitoring accuracy is 61% for 30 people and 68% for 50 people. On the traffic bridge, the monitoring accuracy is 76% for 30 people and 79% for 50 people. The monitoring accuracy of the model can ensure the detection requirements, but in areas with large scope and complex conditions such as football fields and ports, the monitoring accuracy cannot be guaranteed to be more than 70%. Therefore, comprehensively speaking, the monitoring accuracy of the model can meet the ordinary monitoring requirements.

## 4. Conclusions

With the emergence of various new tracking technologies, the combination of computer technology and various electronic detection technologies has become mature. The application of diversified target detection technology in all walks of life has become a popular research direction. For the problems of nonstandard movement and single movement mode, with the video of sports events as the object, a target detection and tracking system model is proposed to track and detect the targets in sports in real-time. Experimental analysis shows that the proposed model can maintain high stability with a loss rate of 3% and a response speed of 4 s and can track and monitor the moving target with 80% target detection accuracy. It is expected to provide reference ideas for the follow-up sports events and postgame video analysis through experiments. However, this exploration also has some deficiencies. For example, the method studied is only a part of the intelligent video surveillance system, and the relevant theoretical research work is insufficient. The system architecture, video codec algorithm, and target recognition are also important components for the specific system. Therefore, it can be considered to be studied from the perspective of the whole intelligent video surveillance system. Next, the proposed model algorithm optimizes the target-dense scene. Hence, the large field of vision, complex background, target scale transformation and deformation will be further studied. Moreover, relevant information mining should be conducted to further optimize the proposed model algorithm and apply it to the detection and analysis of moving targets in actual sports events as soon as possible.

## Figures and Tables

**Table 1:** Image processing strategy in DL.

Image processing mode	Processing scenario
Remote sensing dataset of land use image	According to the centralized calculation of the city map, the calculation scenes are sparse residential areas, traffic channels, and public parking lots.
Remote sensing dataset of Wuhan university	According to the spatial resolution calculation, the analysis scenes are general residential areas, traffic stations, ponds, rivers, and mountainous areas.
Remote sensing image dataset	According to the basic expansion calculation of the school, the scene includes airports, flat plains, baseball stadiums, beaches, and other places.
Remote sensing dataset of space objects	According to the analysis of google maps, the general calculation resolution is high, including docks, industrial storage places, various sports places, ports, bridges, and other areas.

**Table 2:** Target tracking core area.

Core area	Regional representation
Selection criteria of candidate areas	The size and proportion of different areas may vary, so it is essential to search and filter all areas that can match when selecting memory areas. however, this method is time-consuming and laborious, and will produce many property management areas, so feature analysis is a vital link.

Feature representation of candidate regions	After the collective search and analysis of the region, the region's overall candidate region can be obtained. The next step is to filter its characteristics. Given the diversity of factors in the region itself, the feature extraction will affect the final classification results. Therefore, in most cases, the haar-link features are used for processing.

Classification measures of candidate areas	Since different regions will produce different differences in the processing of different objects, the input features can be effectively classified after massive feature processing. support vector machines, decision trees and neural networks are commonly used. by calculating multiple features, a classifier with strong performance can be obtained.

**Table 3:** Three-layer structure requirements for target tracking.

Layers	Hierarchy function requirements
Application layer	The main requirement and function of the application layer is to match the motion detection target with the whole tracking system. Generally, it is for users to access the sports multimedia module, the multimedia video recording module, and the tracking target management module and record data.

Data access layer	The main function of the data layer is to play the role of the middle layer, establish the detection algorithm, and generate data with the detection target in the sports multimedia image or video.

Data connection layer	The function of the connection layer is to store and track the data information generated by the above two layers, multimedia video or image information, user information, and detection results.

## Data Availability

All data are fully available without restriction.
